# Integration of the Metabolomic and Transcriptome Analysis Reveals the Remarkable Compounds of *G. bicolor* Young and Mature Leaves under Different Iron Nutrient Conditions

**DOI:** 10.3390/ijms23031160

**Published:** 2022-01-21

**Authors:** Zhe Feng, Shuyu Ji, Di Cui

**Affiliations:** 1 College of Biosystems Engineering and Food Science, Zhejiang University, 866 Yuhangtang Road, Hangzhou 310058, China; 11713010@zju.edu.cn (Z.F.); jishuyujade@zju.edu.cn (S.J.); 2 Key Laboratory of on Site Processing Equipment for Agricultural Products, 866 Yuhangtang Road, Hangzhou 310058, China

**Keywords:** metabolomics, transcriptome, Fe-deficiency stress, *G. bicolor*, glutamate

## Abstract

*Gynura bicolor* (Roxb. ex Willd.) DC. (*G. bicolor*) is a functional vegetable rich in iron (Fe) and widely grown in Asia (e.g., Japan and China). Because most Fe in the soil exists in the form of insoluble oxides or hydroxides, it is difficult for plants to obtain Fe from the soil. A comparative metabolomic and transcriptome study was carried out to investigate the effect of Fe deficiency on metabolite synthesis and gene expression in young and mature leaves of *G. bicolor*. Fe deficiency caused chlorosis and decreased the chlorophyll content in young leaves. The metabolomic results for young leaves showed that l-glutamate and 4-hydroxybutanoic acid lactone significantly increased and decreased, respectively. The transcriptome results showed that the expression levels of genes involved in ferric reduction oxidase 7 and 14-kDa proline-rich protein DC2.15-like were significantly upregulated and downregulated, respectively. However, Fe deficiency had little effect on mature leaves.

## 1. Introduction

Iron (Fe) is the fourth most abundant element in the Earth’s crust, but it is usually present in the form of insoluble oxides or hydroxides. Especially in saline and alkaline soils, the solubility of Fe is extremely low [1]. Iron is also an essential element for plants and is involved in many important biological processes, such as nitrogen fixation [2], photosynthesis [3] and respiration [4]. Plants have two different strategies to dissolve and transport Fe (strategy 1: H^+^-ATPase, Fe^3+^ reduction, and Fe^2+^ transport system; strategy 2: synthesis and secretion of mugineic acid). In addition, Fe deficiency affects the synthesis of cytochromes, catalases, peroxidases, and metalloflavoproteins. A phenotype of Fe deficiency in plants is obvious; chlorosis occurs because of the inhibition of chlorophyll (Chl) synthesis (leaf chloroplasts contain 80% iron [5]). Interveinal chlorosis first appears in young leaves [5]. Fe deficiency severely affects energy and substance metabolism in plants. Prolonged iron deficiency results in quality deterioration and yield losses of crops. In addition, Fe deficiency in crops can also affect the human intake of Fe, leading to diseases such as iron-deficiency anaemia. Moreover, Fe is one of the most abundant trace elements in the human body, and its main roles are as follows: (1) it is an important component of haemoglobin and is involved in oxygen transport and storage; (2) it has an impact on the immune system; and (3) it is directly involved in metabolism [6]. Adolescents and menstruating women have a high demand for iron [7]. Fortunately, plants can induce a series of response mechanisms to adapt to Fe-deficiency stress. Roots will secrete a lot of organic acids, phenols and other reducing substances, thus increasing the solubility of external Fe [8,9]. Moreover, some plants regulate the metabolism of butanoate and amino acid to enhance tolerance [10].

*Gynura bicolor* (Roxb. ex Willd.) DC. (*G. bicolor*) is a functional plant grown in many countries (including China, Japan, Thailand and Myanmar). *G. bicolor* is well known for its richness in minerals, vitamins, fibre and protein [11]. In particular, its leaves have iron (Fe) levels as high as 3.26 μg/g, and its extracts have been shown to promote the bioavailability of Fe in rats [12]. Moreover, in many countries, *G. bicolor* is used not only as food but also as folk medicine [13]. It has been used customarily as a traditional herbal medicine for dysmenorrhea, iron-deficiency anaemia, and diabetes mellitus, among other conditions [14]. Therefore, an increasing number of plant factories cultivate *G. bicolor*, and its business value will be further explored in the future.

Plant growth conditions are unsuitable, complex and changeable (e.g., nutrition deficiency, chilling and drought stress). They will evolve a series of resistance mechanisms such as gene expression regulation and metabolic release [15,16]. Most mineral deficiencies affect plant hormone secretion, enzyme activity, reaction oxygen metabolism and photosynthesis. For example, zinc deficiency will significantly influence energy metabolism and protein synthesis [17]. Potassium deficiency will lead to the accumulation of putrescine, particularly in leaves, which can be used as the biomarker metabolite to monitor nutritional needs of plants [18]. To deeply understand plant responses under different growth conditions, high-throughput and efficient multi-omics joint analysis technology has been introduced in the field of plant physiology. An increasing number of researchers are using metabolomics and transcriptomics to elucidate the genetic mechanisms of plant responses to abiotic stress [19,20]. Metabolomic analysis can serve as an important supplementary method for transcriptome to maximize the mining of key, hidden information and to validate core data [21]. 

In this study, metabolomic and transcriptome approaches were used to analyze the young and mature leaves of *G. bicolor* under Fe deficiency. The biomarkers and key genes of leaves were examined, and the adaptive mechanisms of *G. bicolor* to Fe deficiency were investigated. An understanding of these mechanisms can serve as a basis for the development of cultivars with Fe-deficiency tolerance.

## 2. Results

### 2.1. Phenotypic Analysis of G. bicolor under Fe-Deficiency Stress

**Figure 1:**
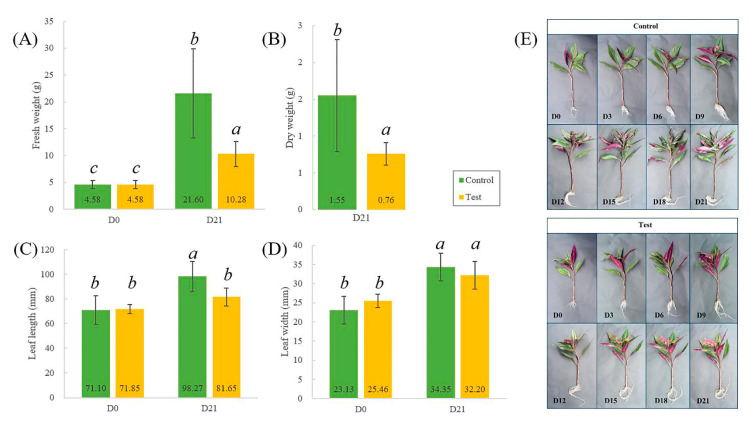
The effect of Fe-deficiency stress on fresh and dry weight (**A**,**B**) and leaf length and width (**C**,**D**). Photographs of *Gynura bicolor* under Fe-deficiency stress (**E**). Bars indicate standard deviation (SD, *n* = 24). Control = 36.7 mg/L FeNaEDTA; Test = 0 mg/L FeNaEDTA. Different letters indicate significant differences, and the same letters indicate nonsignificant differences (ANOVA, *p* < 0.05). Di refers to the abbreviations of Day *i* (*i* = 0, 3, 6, 9, 12, 15, 18 and 21).

**Figure 2:**
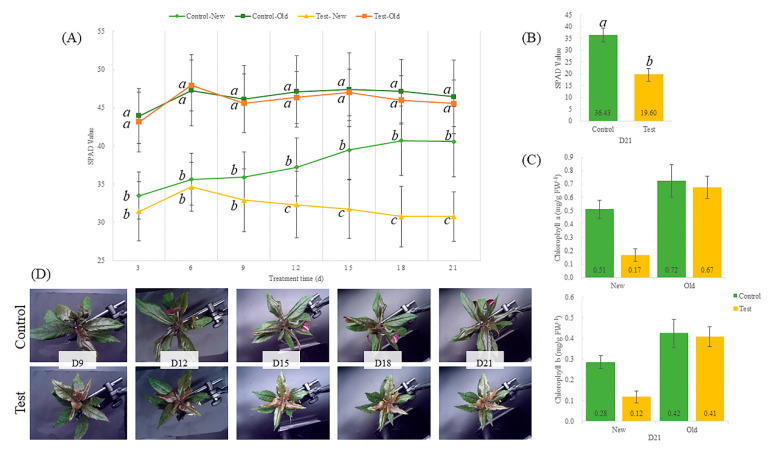
SPAD values of new and old leaves (**A**) measured in the top leaves (leaf length > 3 cm, n = 24) (**B**). Effect of Fe-deficiency treatment on chlorophyll a and b (**C**). Photographs of *Gynura bicolor* under Fe-deficiency stress (**D**). Leaves that grew during this experiment were defined as new leaves, and leaves that existed before this experiment (leaf length > 5 cm) were defined as old leaves. Bars indicate standard deviation (SD). Control = 36.7 mg/L FeNaEDTA; Test = 0 mg/L FeNaEDTA. Different letters indicate significant differences, and the same letters indicate nonsignificant differences (ANOVA, *p* < 0.05). Di refers to the abbreviations of Day *i* (*i* = 0, 3, 6, 9, 12, 15, 18 and 21).

Twenty-four plants were randomly selected for fresh and dry weight determination. Following 21 days of Fe deficiency, there was a significant reduction in both fresh and dry weight of *G. bicolor* compared to the control group (Figure 1). Fe-deficiency treatment also led to a reduction in leaf length, but the effect on leaf width was not significant (n = 10) (Figure 1). The SPAD values of old leaves were not significantly different between the control and test groups during 21 days of treatment (Figure 2). However, the SPAD value of new leaves tended to decrease during the Fe-deficiency treatment, while that of new leaves in the control group increased (Figure 2). Fe-deficiency treatment significantly decreased the SPAD value of the top leaves (length > 3 cm) in the control group on Day 21 (Figure 2). Similarly, Chl a and b in new leaves (test group) were only 0.17 and 0.12 mg/g, while those in the control group were 0.51 and 0.28 mg/g, respectively (Figure 2). Fe deficiency slowed the growth of *G. bicolor* and adversely affected Chl a and b synthesis in new leaves. 

### 2.2. Effect of Fe-Deficiency on Element Content of Leaves

As shown in Table 1, the Fe concentration significantly decreased in new leaves under Fe-deficiency stress, while there was no significant change in old leaves between the two groups. Moreover, the imposition of Fe-deficiency treatment significantly increased the zinc (Zn) content in old and new leaves. The concentration of phosphorus (P) also increased in new leaves. However, there were no significant changes in calcium (Ca) and magnesium (Mg) contents.

**Figure 3:**
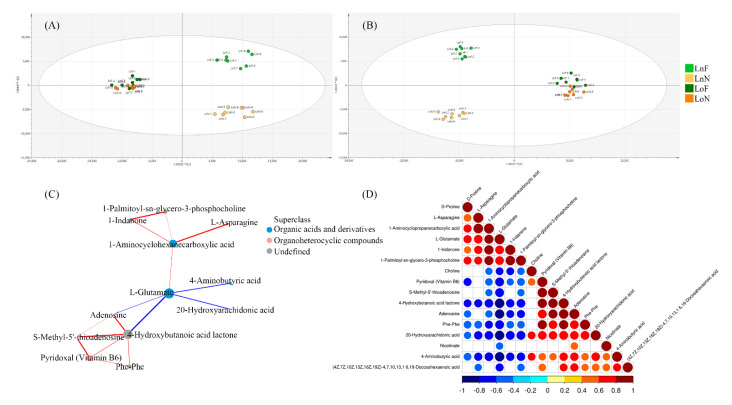
Metabolomics analysis of *Gynura bicolor* leaves in response to Fe deficiency. Orthogonal partial least squares discriminant analysis (OPLS-DA) score plots in the positive (**A**) and negative (**B**) ion modes of *G. bicolor* leaf samples. (**C**) Correlation network of metabolites from the new leaves. Node size and color indicate the degree and classification, respectively. Red lines correspond to a positive correlation, and blue lines correspond to a negative correlation. (**D**) The correlation heatmap was based on the metabolites found in the new leaves. (LoF, the old leaves of the control group; LoN, the old leaves of the test group; LnF, the new leaves of the control group; LnN, the new leaves of the test group).

An untargeted metabolomics approach was used to explore the difference in metabolites between the control and test groups. The OPLS-DA results for the new and old leaves using t[1] × t[2] score plots distinguished the test group from the control group (Figure 3). In the positive and negative ion modes, the control and test groups were separated effectively for new leaves (Figure 3). However, there was no significant difference between the control and test groups for old leaves (Figure 3), and the volcano plots of metabolomics in the nTable ew and old leaf samples were shown in Figure S1. Iron is not readily mobile in different parts of the plant, and this is reflected by chlorosis of the youngest leaves but not the older leaves. The classification results for old leaves showed that Fe deficiency had no significant effect on *G. bicolor* in terms of leaf color, but it also did not significantly affect the synthesis of metabolites.

The identified compounds in leaf samples were mainly organic acids and derivatives, organic oxygen compounds, lipids and lipid-like molecules. Significant changes in metabolites are shown in Table 2, and the number of decreased metabolites in new leaf samples was higher than that of increased metabolites. The metabolites that increased and decreased the most were l-glutamate (an approximately 12-fold increase) and 4-hydroxybutanoic acid lactone (an approximately 8-fold decrease). In addition, 1-aminocyclopropanecarboxylic acid (ACC) and d-proline increased, while 4-aminobutyric acid, nicotinate, pyridoxal (vitamin B6), choline, adenosine and s-methyl-5’-thioadenosine decreased in new leaves in the test group. In contrast to the results for the new leaf samples, cytidine, 2’-O-methyladenosine and adenosine increased, and adenosine decreased in the old leaf samples. Moreover, the number of the changed metabolites in old leaf samples was less than that in new leaf samples. Correlation network analysis results and the correlation heatmap based on the metabolites found in the new leaves were shown in Figure 3, and these results exhibit the relationships between metabolites in the new leaf samples. L-glutamate, 4-hydroxybutanoic acid lactone and 1-aminocyclopropanecarboxylic acid had the high connectivity and l-glutamate had the highest number of connections.

**Figure 4:**
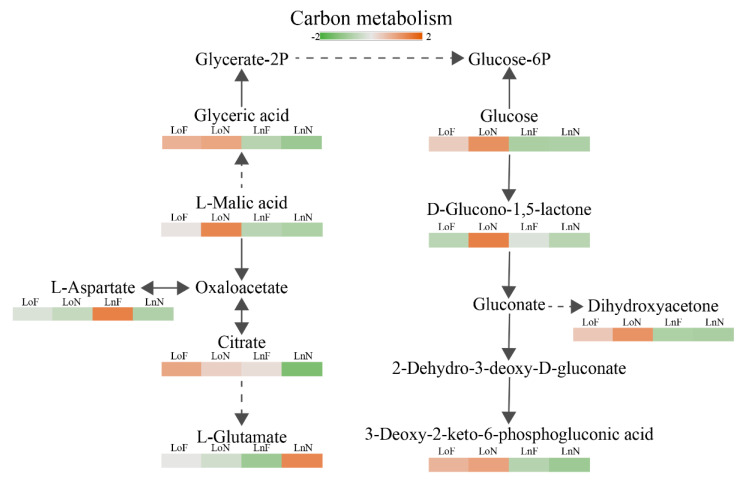
The carbon metabolism pathway shows the changes in *Gynura bicolor* leaves in response to Fe deficiency. Heatmaps show the different accumulations of metabolites. (LoF, the old leaves of the control group; LoN, the old leaves of the test group; LnF, the new leaves of the control group; LnN, the new leaves of the test group.).

The changes of leaf metabolites involved in the carbon metabolism were shown in Figure 4. The contents of l-malic acid, alpha-d-glucose and 3-deoxy-2-keto-6-phosphogluconic acid changed significantly between old and new leaves, while there was no significant difference between the control and test groups of old or new leaves. Moreover, d-glucono-1,5-lactone and dihydroxyacetone had significant changes in the old leaves between the test and control groups, while citrate, l-aspartate, l-glutamate and glyceric acid had significant changes in the new leaves.

### 2.3. Transcriptome Analysis of the G. bicolor Response to Fe Deficiency

**Figure 5:**
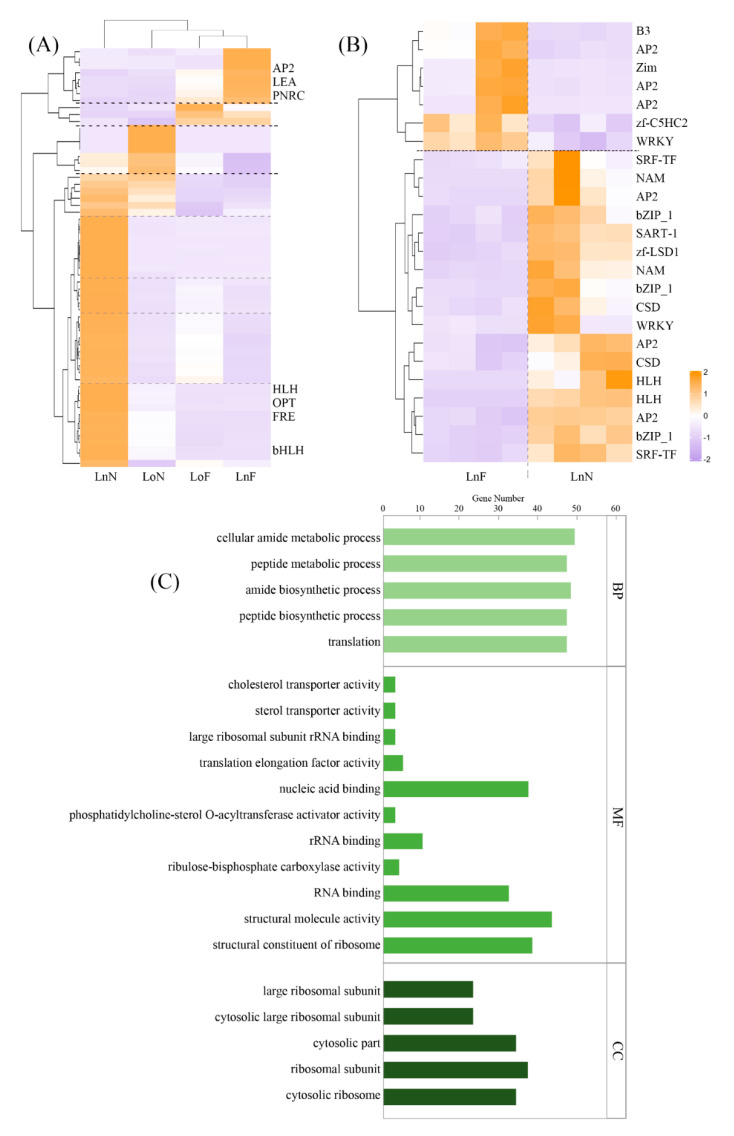
Transcriptome analysis of *Gynura bicolor* leaves in response to Fe deficiency. Heatmap showed the different accumulations of genes in both old and new leaf samples (**A**). Heatmap showed the key different accumulations of genes in new leaf samples (**B**). GO enrichment analysis of the differentially expressed genes in the new leaf samples (**C**). (LoF, the old leaves of the control group; LoN, the old leaves of the test group; LnF, the new leaves of the control group; LnN, the new leaves of the test group).

**Figure 6:**
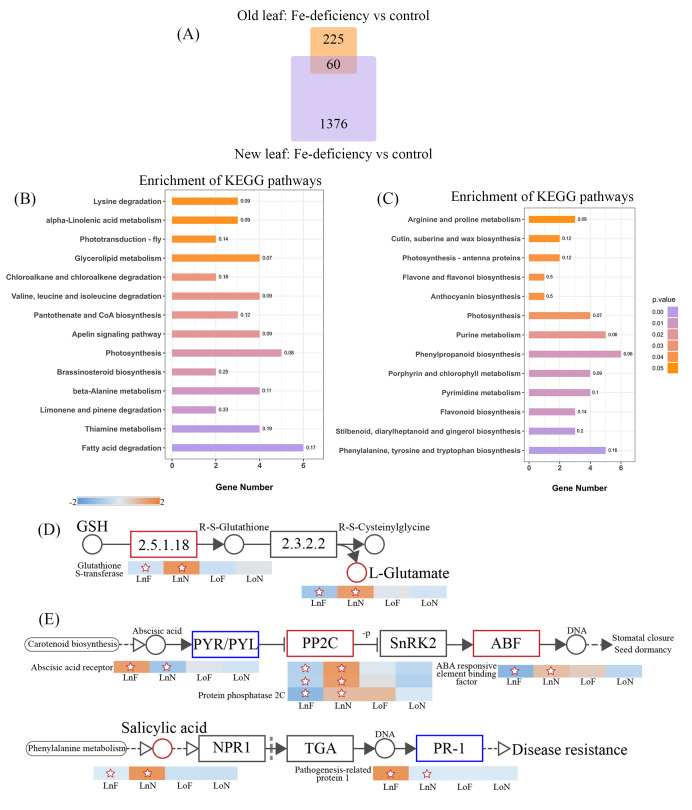
Transcriptome and metabolomic analysis of *Gynura bicolor* leaves in response to Fe deficiency. (**A**) The number of differentially expressed genes (DEGs) in the new and old leaf samples. (**B**,**C**) KEGG pathway enrichment analysis of the DEGs in the new leaf samples ((**B**): upregulated; (**C**): downregulated). (**D**,**E**) Pathway analysis of metabolites and genes in leaves of *G. bicolor* under Fe-deficiency conditions. Different colors indicate metabolite and gene accumulation levels (red: upregulated, blue: downregulated). Heatmaps showed the different accumulations of metabolites or genes, and metabolites or genes with significant differences (*p*-value > 0.05) between the control and test groups are denoted by stars. ((**D**): glutathione metabolism; (**E**): plant hormone signal transduction.).

The GO (Figure 5B) and KEGG (Figure 6C,D) enrichment analyses of the *G. bicolor* leaf response to Fe deficiency were carried out to find the key genes. Volcano plots of expressed genes in the new leaf samples were shown in Figure S2. There were 1436 and 285 differentially expressed genes (DEGs) in the new and old leaf samples, respectively, and 60 identical DEGs (Figure 6), which indicated that Fe deficiency had little effect on the transcription process in old leaves. Among DEGs in new leaf samples, 787 DEGs were upregulated and 649 DEGs were downregulated. Moreover, one of the most upregulated genes was related to ferric reduction oxidase 7 (FRO7, approximately 64-fold), while one of the most downregulated genes was related to 14-kDa proline-rich protein DC2.15-like (PRPDC2.15, approximately 16-fold). FROs have diverse roles in Fe distribution and uptake within plants, and FRO7 is responsible for Fe delivery to chloroplasts [22,23,24]. PRPDC2.15 encodes cell wall structural proteins, and Fan et al. (2014) [25] found that it was downregulated in Arabidopsis under aluminum stress. And the expression level of ethylene-responsive transcription factor (ERF) significantly decreased (more than 64-fold). Dai et al., (2018) [26] found that ERF was downregulated in peanuts under Fe-deficiency stress. In Figure 5A, most of the genes were upregulated in test group of the new leaves. Among these, AP2 (APETALA2) was downregulated in the test group (new leaves), which is widely involved in many biological processes such as growth and development and stress response. But HLH/bHLH (bHLH38, more than 200-fold; bHLH62) transcription regulators were upregulated in test group. The expression of bHLH aided the regulation of Fe-deficiency in plants [27]. In Figure 5B, expression of NAM, bZIP, and HLH transcription factors (TRs) were upregulated. Some AP2 TRs were upregulated and some downregulated. AP2, bHLH, NAC and bZIP TRs are all associated with terpenoid metabolism. They are a multigene family ubiquitous in eukaryotes, which can effectively regulate the biosynthesis of plant secondary metabolites. Overexpressed bZIP can significantly improve the ability of plants to resist abiotic stress [28,29]. In the case of the KEGG pathways of DEGs (Figure 6), the pathways related to brassinosteroid biosynthesis, limonene and pinene degradation, thiamine metabolism, chloroalkane, chloroalkene degradation, and fatty acid degradation were significantly upregulated, while the pathways related to phenylalanine, tyrosine and tryptophan biosynthesis, stilbenoid, diarylheptanoid and gingerol biosynthesis, flavonoid biosynthesis, flavone and flavonol biosynthesis, and anthocyanin biosynthesis were significantly downregulated.

### 2.4. The Correlation between the Metabolome and Transcriptome

As shown in Figure 3 and Figure 4, metabolomic and transcriptome data indicated that Fe deficiency had less effect on old leaves. The expression levels of genes involved in glutathione S-transferase (GST) and the content of l-glutamate increased in new leaf samples (Figure 4E). GSTs play an important role in enhancing plant tolerance of the environment and resistance to injury [30], and a high ratio of GSTs is important for ROS scavenging in plants. Zeng et al. (2015) [31] found that the proteins related to GSTs in the sensitive barley genotype dramatically decreased under potassium deficiency stress, and Yang et al., (2019) [32] found that overexpression of GSTs from *Populus* increased the salt and drought tolerance of Arabidopsis. In addition, glutamate is a core amino acid in plants. It not only participates in the synthesis of various amino acids but also regulates important physiological metabolic processes [33]. *Gynura bicolor* might improve tolerance to Fe deficiency by increasing the expression of GSTs.

The expression levels of genes involved in protein phosphatase 2C (PP2C) and abscisic acid (ABA)-responsive element binding factor (ABF) increased, while those in the ABA receptor PYR/PYL family decreased in new leaf samples (Figure 4F). ABF, a regulatory factor, plays an important role in plant resistance to abiotic stress. Wang et al., (2019) [34] found that ABF played a role in cabbage under calcium deficiency stress, and Dossa et al. (2017) [35] found that ABF was upregulated in sesame under drought stress. In addition, PP2C, a constitutive protein phosphatase with multiple functions, is widely involved in various protein kinase signaling pathways caused by stress as a negative regulator in organisms [36]. PP2Cs are involved in potassium deficiency-triggered signaling [37]. Tiwari et al., (2020) [38] found that protein phosphatase-2c was highly upregulated in potato roots under nitrogen stress. ABA receptors are an important signaling molecule in the ABA signaling pathway in plants, and PYR/PYL proteins can inhibit PP2C activity [39]. In this study, the expression levels of genes involved in ABF were upregulated, indicating that *G. bicolor* might have the ability to fight Fe-deficiency stress.

## 3. Discussion

### 3.1. Impacts of Fe Deficiency on Metabolism

Elemental analysis indicated that Fe-deficiency treatment affected new leaves more than old leaves and that Fe was an immobile nutrient [5]. In addition, Zn might play a key role in resistance to Fe-deficiency stress. Similarly, Arrivault et al., (2006) [40] found that Fe deficiency enhanced the accumulation of Zn in the aboveground organs of Arabidopsis. The influx rate for other metal ions (e.g., Zn^2+^, Mn^2+^ and Cd^2+^) will increase when plants are subjected to Fe-deficiency stress [41].

Figure 3 showed that Fe deficiency mainly affected the synthesis of l-glutamate, citrate, l-aspartate and glyceric acid in the carbon metabolism pathway. The metabolomic results indicated that l-glutamate was the “hub” and played the key role in the network. Glutamate was shown to be involved in the synthesis of a variety of proteins and amino acids related to plant stress resistance. Under stresses, glutamate was metabolized to γ-aminobutyric acid (GABA), which resulted in the regulation of pH balance and redox level [42]. However, the metabolomic results showed that the contents of aminobutyric acid and 4-hydroxybutanoic acid lactone decreased in new leaf samples, while the content of glutamine increased. It indicated that the glutamic acid decarboxylase activity might be inhibited. The polyamine degradation pathway was another pathway to synthesize GABA, which might be restricted. Moreover, ACC is a direct precursor of ethylene biosynthesis, and ethylene is an important plant hormone. In this study, the contents of ACC and SA significantly increased, but there was no change in ethylene content. The results proved that SA (a major plant hormone) accumulated due to Fe deficiency and involved in the regulation of defense response. The accumulation of endogenous SA activated Fe translocation via the transcriptional regulation, mediated by bHLH38 (upregulated in this study), of the downstream Fe gene [43]. In summary, *Gynura bicolor* might prefer to stimulate systemic acquired resistance by releasing SA.

The results also showed that the contents of citrate, l-aspartate and glyceric acid decreased in the test group. It agreed with the findings about other abiotic stresses. Urbanczyk-Wochniak & Fernie (2005) [44] found that citrate and aspartate decreased in tomato leaves under nitrate stress and nitrogen deficiency, respectively. And, L. Liu & Lin (2020) [45] also found that citrate decreased in *Sargassum fusiform* leaves under heat stress. However, plant roots synthesize more citrate to cope with the lack of mineral nutrients [46], which might lead to the decline in citrate in leaves. Roots absorb Fe by citrate and Fe complexation, and Fe-citrate is distributed to the mature leaves through transpiration [47]. The photosynthesis and transpiration rate were weakened by Fe deficiency, which might lead to the decline in citrate and some organic acid by influencing the leaf energy metabolism and the tricarboxylic acid cycle.

### 3.2. Impacts of Fe Deficiency on Transcriptome

*Gynura bicolor* might improve tolerance to Fe deficiency by increasing the expression of GSTs. And, the results of transcriptome analysis showed that bHLH TRs might play a key role in Fe-deficiency stress, and the expression of bHLH TRs in new leaves was significantly higher than that in old leaves. What’s more, the effect of Fe-deficiency on transcription of new leaves was greater than that of old leaves. The decline in the expression of ZIP-related TRs in new leaf samples might lead to the drop in Zn content. The expression level of genes involved in pathogenesis-related protein 1 (PR-1) decreased, and PR-1 has biological functions associated with defense against biotic stresses [48]. In this study, SA content increased, but the expression of PR-1 was downregulated, indicating that Fe deficiency would weaken the function in resistance to pathogen attack. Moreover, the expression of bHLH and FRO was upregulated, which indicated that the positive regulatory network of Fe-deficiency-centered FIT (HLH29) transcription regulator was activated [49]. FIT overexpression alone does not enhance the iron deficiency response, but co-expression with bHLH activates iron uptake genes. Co-overexpression of FIT and bHLH38 can activate Fe uptake genes to improve Fe deficiency tolerance. The transcriptome results showed that *Gynura bicolor* could fight against Fe-deficiency stress.

## 4. Materials and Methods

### 4.1. Plant Materials

In this experiment, cuttings were used to propagate *G. bicolor* seedlings from Wuhan Happy Farm Gardening Co., Ltd. Tender stems (similar in length and quality) were selected from healthy parental plants with the same growth as cuttings. The cuttings were cultivated in perlite for two weeks and then moved to hydroponic tanks in an artificial climate chamber (16 h light at 25 ± 1 °C, 8 h dark at 19 ± 1 °C, 70% relative humidity, 300 μmol m^−2^ s^−1^ photon flux density). One hundred and sixty-eight plants were randomly and equally divided into two groups and grown for 21 days in intermittently aerated nutrient solution. Fe-sufficient plants (control group) were grown in normal nutrient solution (consisting of 945 mg/L Ca(NO_3_)_2_·4H_2_O, 506 mg/L KNO_3_, 80 mg/L NH_4_NO_3_, 136 mg/L KH_2_PO_4_, 241 mg/L MgSO_4_, 36.7 mg/L FeNaEDTA, 0.83 mg/L KI, 6.2 mg/L H_3_BO_3_, 16.9 mg/L MnSO_4_·H_2_O, 8.6 mg/L ZnSO_4_·7H_2_O, 0.25 mg/L Na_2_MoO_4_·2H_2_O, 0.025 mg/L CuSO_4_·5H_2_O, and 0.025 mg/L CoCl_2_·6H_2_O) and Fe-deficient plants (test group) were grown under iron-deficient conditions (normal nutrient solution without FeNaEDTA). The first pair of leaves from top to bottom and the first pair from bottom to top were marked (leaf length > 3 cm). On Day 0 and Day 21, fresh weight (FW), leaf length and leaf width were recorded. SPAD (Konica Minolta, SPAD-502 Plus, Tokyo, Japan) readings were made every two days. Leaves that grew during this experiment (young leaves) were defined as new leaves, and those that existed before this experiment (mature leaves, leaf length > 5 cm) were defined as old leaves. The plants were placed in a drying oven at 105 °C for 15 min, and then the temperature was adjusted to 70 °C until a constant weight was reached. The dry weight (DW) was then measured. Microwave digestion and inductively coupled plasma–optical emission spectrometry (ICP-OSE) was used for trace element analysis (Fe, Mg, Zn, P and Ca) in plants. Forty-eight plants were randomly selected for metabolomics analysis (twenty-four of these were used for transcriptome analysis). These samples were powdered in liquid nitrogen and stored at −80 °C.

### 4.2. Determination of Chlorophyll Content

Leaf samples were collected on Day 21. Samples were weighed to 1 g and then extracted with 80% acetone solution for 30 min in a dark environment. Under light-shielding conditions, the extract was filtered, and the absorbance was measured using a UV-Visible spectrophotometer (Thermo Scientific, Evolution 220). Chlorophyll a and b contents (mg/g) were calculated using the following formula [50,51]:

(1) $$C_{a}=(12.71{\times A}_{663}-2.59{\times A}_{645})\times V/W$$

(2) $$C_{b}=(22.88{\times A}_{645}-4.67{\times A}_{663})\times V/W$$

where C_a_ and C_b_ are the amounts of chlorophyll a and b, respectively. A663 and A645 were the absorbance values at 663 and 645 nm, respectively. V was the volume of the extract, and W was the FW of the sample.

### 4.3. Transcriptome Data Acquisition

The leaves of each plant were removed from the same position. The first pair of new leaves (leave length > 3 cm) on the top and the last pair of old leaves at the bottom were removed from each plant (Figure S3). RIzol^®^ Reagent was used to extract total RNA from the leaves according to the manufacturer’s instructions (Magen). A Nanodrop ND-2000 system (Thermo Scientific, CA, USA) was used to detect the A260/A280 absorbance ratio of RNA samples. The overall quality of the samples was determined by agarose gel electrophoresis. An ABclonal mRNA-seq Lib Prep Kit (ABclonal, Shanghai, China) was used to prepare the paired-end libraries. The fragmentation of mRNA (purified from 1 μg total RNA) was carried out in ABclonal First Strand Synthesis Reaction Buffer. Subsequently, first- and second-strand cDNAs were synthesized (mRNA fragments as templates used random hexamer primers and RNase H, DNA polymerase I, dNTPs and buffer) and were used for PCR amplification. An Illumina NovaSeq 6000 instrument was used for sequencing.

### 4.4. Metabolomics Data Acquisition

Frozen samples were mixed with methanol/acetonitrile/aqueous solution (2:2:1, *V*/*V*). Quality control (QC) samples (mixing 10 μL of each sample) were prepared to ensure the reliability of instrument analysis. An ultra-performance liquid chromatography system (1290 Infinity LC, Agilent Technologies) coupled to quardruple time-of-flight (AB Sciex TripleTOF 6600) was used for metabolomics analysis. Samples were analyzed by HILIC separation (column temperature: 25 °C, flow rate: 0.3 mL/min). The mobile phase contained Phase A (25 mM ammonium acetate and 25 mM ammonium hydroxide in water) and Phase B (acetonitrile). Chromatographic and ESI source conditions are described in the Supplemental Material.

### 4.5. Data Analysis

Statistical analyses were performed using SPSS Statistics 19 software (IBM, Chicago, USA). Data were shown as averages. According to the results of the variance homogeneity test, a one-way analysis of variance (ANOVA) and independent samples t-test were used to detect differences. A probability (*p*) value of <0.05 was considered statistically significant. Orthogonal partial least squares discriminant analysis (OPLS-DA) was used to differentiate the leaf samples by SIMCA 13.0 software (Umetric, Umea, Sweden). The variable importance for the projection (VIP) obtained from the OPLS-DA model can be used to measure the strength of the expression pattern of each metabolite on the classification of each group of samples. In this experiment, VIP > 1 and a *p*-value < 0.05 were used as the screening criteria for significantly different metabolites. The differentially expressed genes were adopted by KEGG enrichment analysis.

## 5. Conclusions

In this study, the molecular mechanism of *G. bicolor* response to Fe deficiency was investigated by combined metabolomics and transcriptome analyses. The effects of Fe-deficiency stress on the new and old leaves of *G. bicolor* were also studied. Fe deficiency can lead to the yellowing of young leaves of plants but has no significant effect on the color of mature leaves, findings that were verified in this study. In addition, the effect of Fe deficiency on metabolism and transcription in mature leaves was less than that in young leaves. Our results indicate that Fe deficiency mainly influenced glutathione metabolism and plant hormone signal transduction in young leaves, which suggests that these pathways play a key role in the response to Fe-deficiency stress in *G. bicolor*. Furthermore, Fe deficiency resulted in significant downregulation of the pathways related to flavonoid biosynthesis, flavone and flavonol biosynthesis, and anthocyanin biosynthesis, which might lead to a decrease in the nutritional value of *G. bicolor*. Moreover, *G. bicolor* showed active resistance to Fe-deficiency stress; its protein interaction relationships can be further explored in future research.

## Figures and Tables

**Table 1:** The element content of leaves of *Gynura bicolor* in different Fe treatments.

Tissue	Group	Element Content (mg/g FW-1)				
		Fe	Ca	Mg	Zn	P
New leaf	Control	0.04 ± 0.022	16.25 ± 2.56	7.57 ± 0.94	0.06 ± 0.00	4.94 ± 0.40
	Test	0.02 ± 0.006 *	18.21 ± 1.81 ns	7.49 ± 0.52 ns	0.16 ± 0.02 **	6.46 ± 0.52 **
Old leaf	Control	0.06 ± 0.017	11.85 ± 1.16	5.46 ± 0.36	0.06 ± 0.01	4.16 ± 0.20
	Test	0.04 ± 0.021 ns	12.82 ± 2.62 ns	5.52 ± 1.12 ns	0.11 ± 0.03 **	4.90 ± 1.08 ns

Note: The values show mean ± SD from ten replicates. An independent samples *t* test was used to calculate *p* values. * *p* < 0.05, ** *p* < 0.01, ns = not significant. Control = 36.7 mg/L FeNaEDTA; Test = 0 mg/L FeNaEDTA.2.3. Metabolomics Analysis of G. bicolor Response to Fe Deficiency.

**Table 2:** Characteristics of differential metabolites of *Gynura bicolor* in different Fe treatments. (n = 8).

Iron Mode	Tissue	Metabolites	VIP	Fold Change	*p*-Value	*m*/*z*	RT(s)
Positive	New leaf	4-Hydroxybutanoic acid lactone	1.57	0.11	0.0000	87.0432	402.47
		Phe-Phe	1.00	0.23	0.0000	313.1533	154.17
		Pyridoxal (Vitamin B6)	2.10	0.36	0.0000	168.0639	95.96
		4-Aminobutyric acid	2.00	0.37	0.0000	104.0705	368.79
		S-Methyl-5’-thioadenosine	3.65	0.38	0.0000	298.0953	98.92
		Adenosine	13.85	0.47	0.0001	268.1026	168.71
		Nicotinate	1.04	0.56	0.0415	124.0373	215.08
		Choline	1.58	0.59	0.0458	104.1059	390.71
		20-Hydroxyarachidonic acid	1.19	0.64	0.0000	343.2246	34.76
		(4Z,7Z,10Z,13Z,16Z,19Z)-4,7,10,13,16,19-Docosahexaenoic acid	1.41	0.66	0.0137	328.2463	68.75
		D-Proline	1.57	1.92	0.0361	116.0693	308.51
		L-Asparagine	1.86	3.30	0.0001	133.0592	396.60
		1-Indanone	1.24	3.58	0.0035	193.0843	32.62
		1-Aminocyclopropanecarboxylic acid	1.14	3.62	0.0002	84.0433	376.98
		1-Palmitoyl-sn-glycero-3-phosphocholine	1.02	7.70	0.0085	496.3372	188.96
		L-Glutamate	1.99	12.71	0.0000	148.0591	394.20
	Old leaf	Adenosine	4.36	0.46	0.0025	268.1026	168.71
		Cytidine	2.74	2.10	0.0074	244.0922	236.66
		2’-O-methyladenosine	1.46	1.48	0.0154	282.1183	130.78
		Uracil	1.02	1.22	0.0226	113.0331	162.04
Negative	New leaf	Salicylic acid	1.00	2.76	0.0001	137.0237	78.27
		Citrate	3.58	0.06	0.0004	191.0203	470.66
		L-Glutamine	1.08	3.71	0.0009	145.0609	386.60
	Old leaf	D-Lyxose	1.66	1.51	0.0072	149.0445	153.51
		D-Glucono-1,5-lactone	1.28	1.94	0.0185	177.0394	158.68

## Data Availability

The data presented in this study are available on request from the corresponding author. The data are not publicly available due to privacy.

## Supplementary Materials

The following supporting information can be downloaded at: https://www.mdpi.com/article/10.3390/ijms23031160/s1.
