# Supplementary material for: Integration of the Metabolomic and Transcriptome Analysis Reveals the Remarkable Compounds of G. bicolor Young and Mature Leaves under Different Iron Nutrient Conditions
Source: Int J Mol Sci. 2022 Jan 21;23(3):1160. doi: 10.3390/ijms23031160 (PMC8835294; doi:10.3390/ijms23031160)
Supplement: Supplementary file 1 [file ijms-23-01160-s001.zip › ijms-1538448-supplementary.pdf]

Supplemental File

Chromatographic and ESI source conditions:

For HILIC separation, samples were analyzed using a 2.1 mm × 100 mm ACQUIY UPLC BEH 1.7 µm column (waters, Ireland). In both ESI positive and negative modes, the mobile phase contained A=25 mM ammonium acetate and 25 mM ammonium hydroxide in water and B= acetonitrile. The gradient was 85% B for 1 min and was linearly reduced to 65% in 11 min, and then was reduced to 40% in 0.1 min and kept for 4 min, and then increased to 85% in 0.1 min, with a 5 min re-equilibration period employed.

The ESI source conditions were set as follows: Ion Source Gas1 (Gas1) as 60, Ion Source Gas2 (Gas2) as 60, curtain gas (CUR) as 30, source temperature: 600℃, IonSpray Voltage Floating (ISVF) ± 5500 V. In MS only acquisition, the instrument was set to acquire over the m/z range 60-1000 Da, and the accumulation time for TOF MS scan was set at 0.20 s/spectra. In auto MS/MS acquisition, the instrument was set to acquire over the m/z range 25-1000 Da, and the accumulation time for product ion scan was set at 0.05 s/spectra. The product ion scan is acquired using information dependent acquisition (IDA) with high sensitivity mode selected. The parameters were set as follows: the collision energy (CE) was fixed at 35 V with ± 15 eV; declustering potential (DP), 60 V (+) and −60 V (−); exclude isotopes within 4 Da, candidate ions to monitor per cycle: 10.

Supplemental Figure

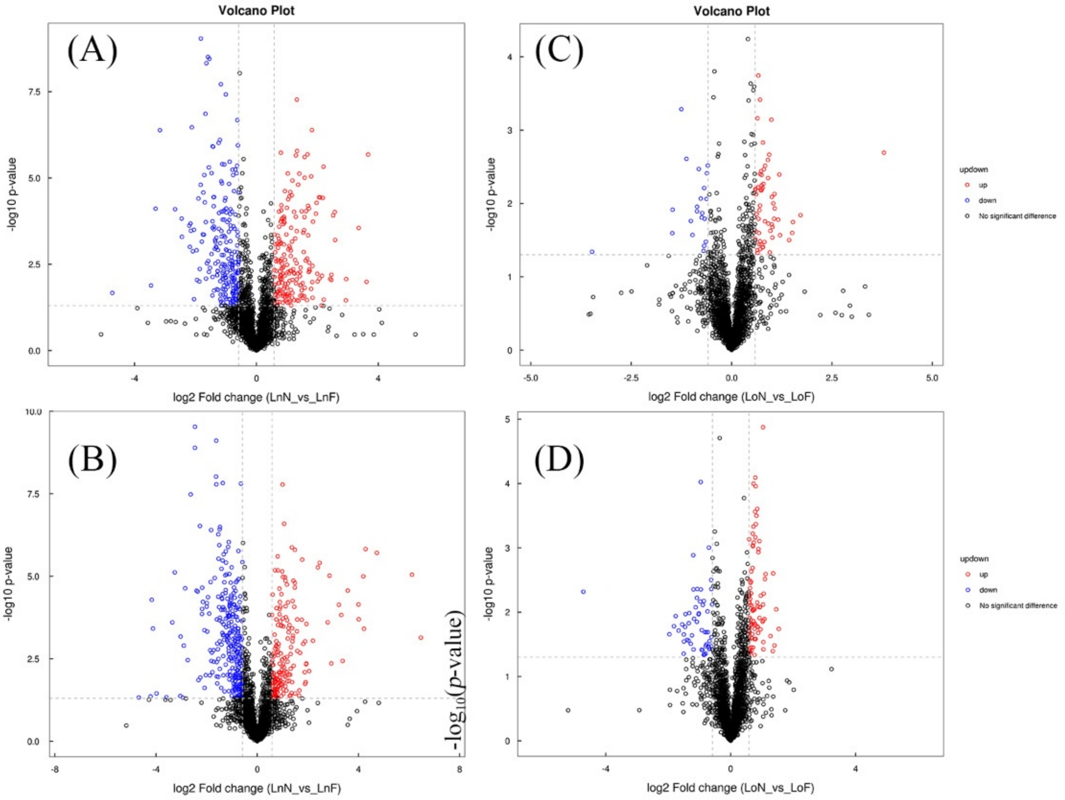

Figure. S1 Volcano plot of metabolomics in the new leaf samples (A: in the positive ion mode; B: in the negative ion mode) and old leaf samples (C: in the positive ion mode; D: in the negative ion mode).

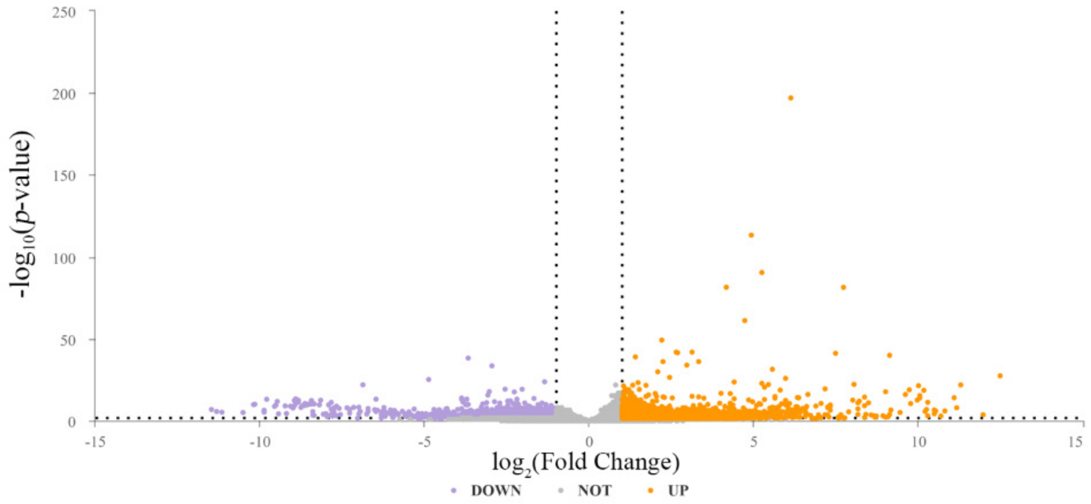

Figure. S2 Volcano plot of expressed genes in the new leaf samples.

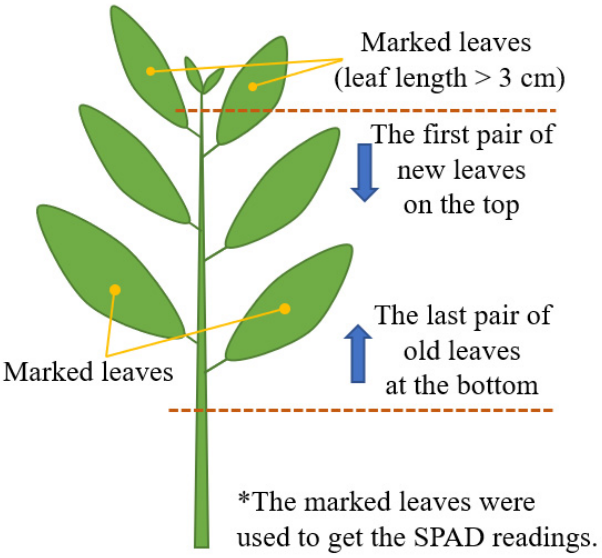

Figure. S3 Diagram of leaf sample acquisition.
